# Preparation and Properties of Polyaniline/Hydroxypropyl Methylcellulose Composite Conductive Thin Films

**DOI:** 10.3390/ma17112687

**Published:** 2024-06-02

**Authors:** Xu Cao, Yinqiu Wang, Yu Zhang, Zenghui Qian, Guodong Jiang

**Affiliations:** College of Materials Science and Engineering, Nanjing Tech University, Nanjing 211816, China; 202161103076@njtech.edu.cn (X.C.); 202261203272@njtech.edu.cn (Y.W.); 202362032047@njtech.edu.cn (Y.Z.); 202361203160@njtech.edu.cn (Z.Q.)

**Keywords:** polyaniline, hydroxypropyl methylcellulose, conductive film, graft polymerization

## Abstract

In this work, a chemical grafting polymerization method was employed to synthesize EHPMC-g-PANI self-supporting films. Polyaniline (PANI) was grafted onto hydroxypropyl methylcellulose (HPMC) modified with epichlorohydrin (EPHMC) to obtain an EHPMC-g-PANI aqueous dispersion, which was subsequently dried to form the self-supporting films. The introduction of HPMC, with its excellent film-forming ability and mechanical strength, successfully addressed the poor film-forming ability and mechanical properties intrinsic to PANI. Compared to in situ polymerized HPMC/PANI, the EHPMC-g-PANI exhibited significantly improved storage stability. Moreover, the fabricated EHPMC-g-PANI films displayed a more uniform and smoother morphology. The conductivity of all the films ranged from 10^−2^ to 10^−1^ S/cm, and their tensile strength reached up to 36.1 MPa. These results demonstrate that the prepared EHPMC-g-PANI holds promising potential for applications in various fields, including conductive paper, sensors, and conductive inks.

## 1. Introduction

Polyaniline (PANI) stands out as a highly promising conductive polymer with industrial applications, due to its excellent conductivity, environmental stability, straightforward synthesis, and low-cost raw materials [1,2,3]. Consequently, PANI’s use in metal anticorrosive coatings, conductive coatings, films, electromagnetic shielding, sensors, and electrochromic materials has garnered significant interest [4,5,6,7]. However, the rigid molecular chain and strong interchain forces of PANI result in poor solubility, processability, and mechanical properties, hindering its practical application [8,9]. To address these limitations, extensive research has focused on emulsion polymerization, doping with organic or inorganic protonics, derivative substitution, and composite material modification [10,11,12]. Jiao et al. prepared polyaniline nanotubes using sodium dodecyl sulfate as the surfactant and 3,5-dinitrobenzoic acid as the doping acid, with a conductivity of 2.05 × 10^−2^ S/cm [13]. N. A. Miraqyan et al. synthesized polyaniline derivatives by polymerization of o-anisidine and m-anisidine using ammonium persulfate as the oxidant in a mixture of glacial acetic acid and methanol. The resulting polyaniline derivatives exhibited good solubility in organic solvents [14]. S. Atia et al. prepared a composite material of polyaniline and polyvinyl alcohol, which exhibited complete compatibility [15]. Among these, the composite modification method stands out. Unlike other methods, it not only achieves a synergistic effect among the different components and allows for the flexible design of new material properties, but also enhances the composite material’s mechanical strength through interfacial interaction [16].

Hydroxypropyl methylcellulose (HPMC) is a water-soluble cellulose derivative derived from natural cellulose, one of the most abundant organic renewable resources on Earth [17,18]. HPMC has been widely utilized in various fields, including coatings, pharmaceuticals, food, cosmetics, and electronic products [19,20,21,22]. The development of cellulose-based composite conductive films has benefited greatly from recent research advancements, especially in the area of incorporating various conductive fillers [23]. Hua et al. developed an rGO/AgNP-CNF film based on silver nanoparticles (AgNPs), cellulose nanofibers (CNF), and reduced graphene oxide (rGO), which showcased strong antibacterial effects and enhanced electrical conductivity [24]. L.M. Al-Harbi et al. developed a poly(ethylene oxide)/carboxymethyl cellulose nanocomposite film incorporated with ZnO and GO nanoparticles for applications in advanced electronic devices [25]. Huang et al. prepared a conductive paper based on polyaniline and cellulose nanocrystals through lotion polymerization, with a conductivity of 4.0 S/m [26]. Compared to other fillers, polyaniline offers the distinct advantages of adjustable conductivity and unique chemical characteristics. HPMC’s excellent film-forming ability, mechanical strength, and ability to effectively stabilize PANI in solution make it an ideal substrate for PANI. The combination of HPMC and PANI offers a promising material with diverse potential applications.

Previous research on cellulose and polyaniline has primarily focused on in situ polymerization or blending methods. This study utilized a grafting polymerization approach to enhance the electrical and mechanical properties of the resulting film. This work employed graft copolymerization to synthesize comb-shaped EHPMC-g-PANI composite conductive films, aiming to enhance the film-forming capability and mechanical strength of polyaniline. Water-soluble HPMC was utilized for its abundant reactive hydroxyl groups. Some of these groups were substituted with epoxy groups, which then reacted with aniline to incorporate it into the HPMC molecular chain. Subsequent oxidative polymerization led to the formation of composite conductive thin films via the solution casting method. The chemical composition and microstructure of the composite conductive films were investigated using FT-IR, UV-VIS, and SEM. The thermal properties of the composite conductive films were studied using TG and DSC. The films’ electrochemical and mechanical properties were also measured.

## 2. Experimental Section

### 2.1. Raw Materials

HPMC (η = 50 mPa·s) was purchased from Shanghai Aladdin Technology Co., Ltd. (Shanghai, China); epichlorohydrin (ECIP, 99.9%), hydrochloric acid (HCl, 38wt%), and ammonium persulphate (APS, 99.9%) were purchased from National Pharmaceutical Group Chemical Reagent Co., Ltd. (Shanghai, China); NaOH (99%) was purchased from Xilong Chemical Co., Ltd. (Shantou, China); and aniline (An, 99%) was purchased from Shanghai Macklin Biochemical Co., Ltd. (Shanghai, China) and pre-distilled before use.

### 2.2. Preparation of EHPMC-g-PANI and HPMC/PANI Dispersions

**Figure 1:**
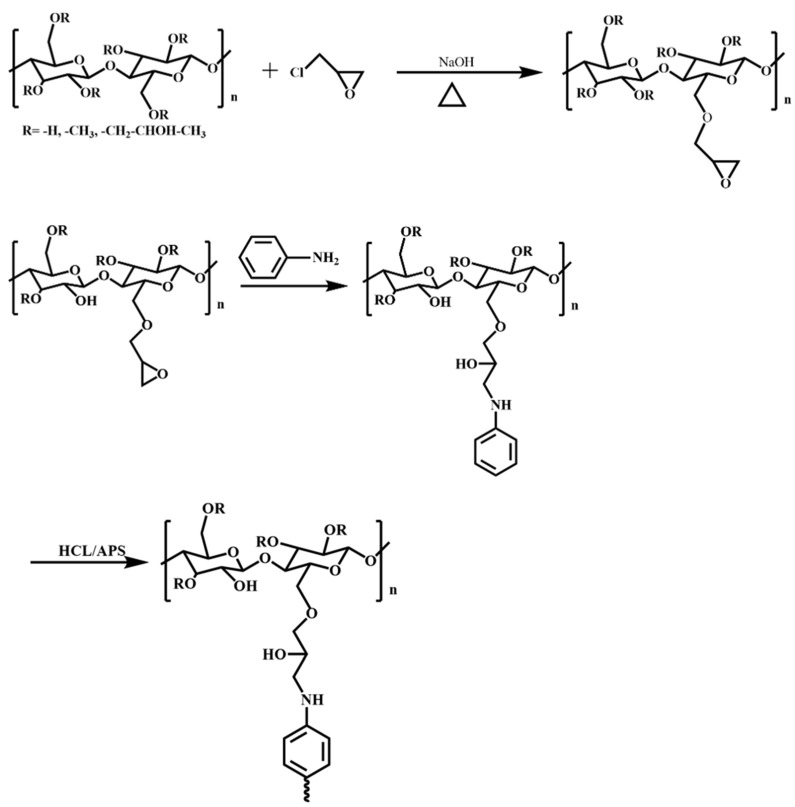
Synthesis pathway of EHPMC-g-PANI.

**Figure 2:**
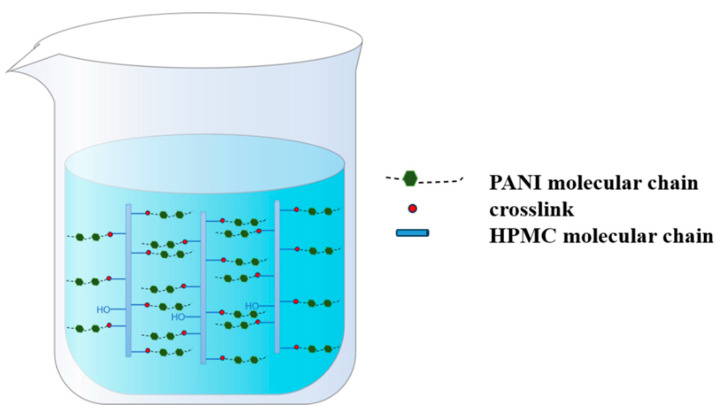
Schematic diagram of molecular dispersion of EHPMC-g-PANI.

Initially, 5.00 g HPMC and 100 mL deionized water were added to a 250 mL flask equipped with a condenser, nitrogen atmosphere, and magnetic stirrer. Then, the mixing was carried out at 25 °C for 30 min to ensure that the HPMC was fully dissolved. Subsequently, the system was heated to 75 °C, and an aqueous sodium hydroxide solution was introduced to adjust the pH to 12. A measured amount of ECIP (20%, 30%, 40%, 50%, and 60% of HPMC weight) was then added dropwise using a constant pressure dropping funnel while continuously monitoring the pH. Once the pH stabilized, the reaction was allowed to proceed for an additional hour, yielding epoxy-grafted hydroxypropyl methyl cellulose. When the reaction was heated up to 80 °C, 1.50 g aniline was added into the flask for a 2 h reaction period, which aimed to introduce the aniline into the molecular chain of the HPMC. Afterwards, the flask was placed in a cryogenic bath at about 0 °C. Next, 7.59 g HCl (38 wt%) and 3.68 g APS (dissolved in 10 mL 0.75 mol/L HCl solution) were added into the flask sequentially and reacted for 24 h. Once the reaction was complete, the obtained product was transferred into a dialysis bag with a molecular weight cutoff of 1000 and dialyzed against deionized water for 24 h, yielding an EHPMC-g-PANI dispersion. The synthesis pathway of the EHPMC-g-PANI is shown in Figure 1. The molecular dispersion of the EHPMC-g-PANI composite conductive material is shown in Figure 2.

The HPMC/PANI dispersion was prepared through in situ polymerization using a similar procedure as mentioned above, where the step of grafting the epoxy groups was omitted.

### 2.3. Preparation of EHPMC-g-PANI and HPMC/PANI Films

The films were prepared by the solution casting method. The purified EHPMC-g-PANI and HPMC/PANI dispersions after dialysis were weighed and casted onto a clean glass plate, which was placed in a blast drying oven at 50 °C. The films were then dried at 50 °C. After the films were completely dried, they were peeled off from the glass plate to obtain the EHPMC-g-PANI composite conductive films.

## 3. Measurements

FT-IR (Nicolet 470, Thermo Fisher Scientific Ltd., Waltham, MA, USA) was used to record the spectrogram of the thin films at frequencies of 2500 to 500 cm^−1^ at a 1 cm^−1^ resolution. The samples were compressed into KBr disks.

NMR (ADVANCE III, Bruker Co., Ltd., Billerica, MA, USA) was used to measure the ^1^H-NMR spectrum of the HPMC and EHPMC-g-PANI films. The solvent was deuterated dimethyl sulfoxide (D-DMSO) and tetramethylsilane was used as the internal standard.

UV-vis (UV-1100, MAPADA Co., Ltd., Shanghai, China) was used to measure the UV-vis spectra of the EHPMC-g-PANI dispersion. The EHPMC-g-PANI dispersion was diluted with deionized water to a concentration of 0.2‰ (*w*/*v*). The UV-Vis absorbance of the EHPMC-g-PANI dispersion was measured using deionized water as the reference solution.

SEM (SM-6510, Nippon Electron Co., Ltd., Tokyo, Japan) was used to examine the surfaces of the composite films. All the samples were gold-coated before testing. The imaging mode used for the test was the secondary electron (SE) mode.

TGA (TGA500, TA Instruments Ltd., New Castle, DE, USA) was used to analyze the thermal properties of the films. The test conditions included a N_2_ atmosphere, a temperature range of 50–800 °C, and a heating rate of 10 °C/min.

DSC (Q200, TA Instruments Ltd., USA) was used to determine the glass transition temperature (T_g_) of the films. Under a nitrogen atmosphere, the samples were heated from 25 °C to 250 °C at a rate of 20 °C/min, held at 250 °C for 5 min, cooled to −10 °C at a rate of 20 °C/min, and finally heated again to 250 °C at a rate of 20 °C/min.

A tour-probe resistivity tester (HPS2663, Changzhou Hailpa Co., Ltd., Changzhou, China) was used to analyze the conductivity of the films.

The tensile strength and elongation at break of the films were measured using a universal testing machine (CMT2103, Meters Industrial Co., Ltd., Shanghai, China), in accordance with GB/T 13022-1991 [27]. The testing speed was set at 10 mm/min, and each sample group was measured at least five times to obtain an average value.

## 4. Results and Discussion

**Figure 3:**
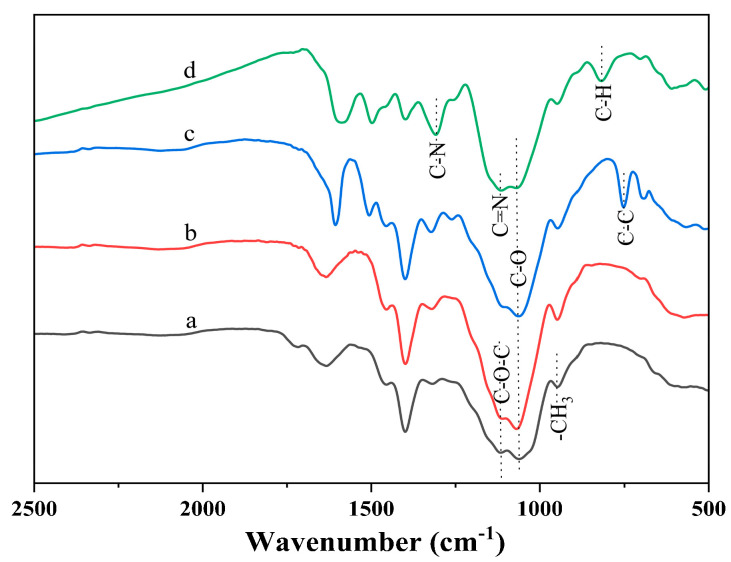
FT-IR spectra for (a) HPMC; (b) EHMC; (c) EHPMC-An; and (d) EHPMC-g-PANI.

To elucidate the molecular structure of the EHPMC-g-PANI, FT-IR was employed to analyze the HPMC, EHPMC, EHPMC-An, and EHPMC-g-PANI following purification (Figure 3). The amount of ECIP in all the samples was 40% by weight of HPMC. The HPMC spectrum exhibited characteristic absorption peaks at 930 cm^−1^, 1316 cm^−1^, 1399 cm^−1^, and 1450 cm^−1^, corresponding to the in-plane and out-of-plane bending vibrations, as well as the symmetric and asymmetric deformation vibrations of the -CH_3_ group, respectively [28]. Upon the introduction of epoxy groups into the EHPMC, a significant intensification of the peak at 930 cm^−1^ was observed, due to overlapping with the epoxy group’s characteristic absorption, along with the enhancement of the C-O absorption at 1059 cm^−1^ and C-O-C absorption at 1114 cm^−1^. The subsequent incorporation of aniline in the EHPMC-An resulted in a decrease in intensity at 930 cm^−1^, 1059 cm^−1^, and 1114 cm^−1^, coupled with the emergence of a new peak at 748 cm^−1^, indicative of C-C bending vibrations within the benzene ring. This confirms the successful integration of aniline molecules into the HPMC chains. Finally, the EHPMC-g-PANI spectrum displayed key peaks at 1503 cm^−1^ (benzene ring backbone vibration), 1127 cm^−1^ (C=N stretching in the quinone ring), and 809 cm^−1^ (C-H out-of-plane bending in the 1,4-substituted benzene ring), confirming the presence of PANI [29]. In summary, PANI was successfully grafted into the molecular chain of HPMC through the action of epichlorohydrin, which was also able to be further verified by NMR analysis.

**Figure 4:**
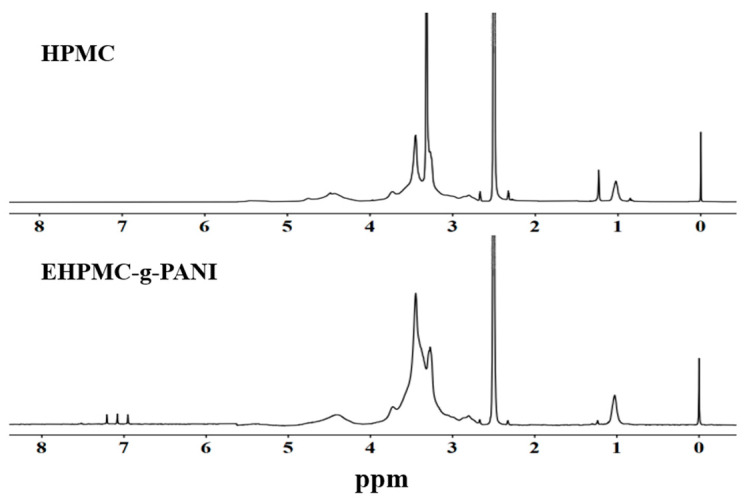
^1^H-NMR spectra for HPMC and EHPMC-g-PANI.

The ¹H-NMR spectra for the HPMC and EHPMC-g-PANI films are presented in Figure 4. The experiment employed D-DMSO as the deuterated solvent, with the peaks at δ = 2.50 ppm and δ = 3.33 ppm corresponding to the solvent and residual water protons, respectively. In the HPMC spectrum, the peaks observed in the region of δ3.3–3.8 ppm were attributed to the protons of the methoxy and hydroxypropyl groups, while the triplet at δ4.4–4.8 ppm represented the hydroxyl protons [30]. Notably, the EHPMC-g-PANI spectrum revealed the transformation of this hydroxyl triplet at δ4.4–4.8 ppm into a broad singlet. This alteration was ascribed to the emergence of amine proton signals in this region following the grafting of PANI. Additionally, new peaks appeared between δ6.9–7.3 ppm, corresponding to the protons of the aromatic and quinoid rings [31]. These characteristic peaks provide evidence that PANI was successfully grafted onto the side chains of HPMC, corroborating the findings obtained from the infrared spectroscopy analysis.

**Figure 5:**
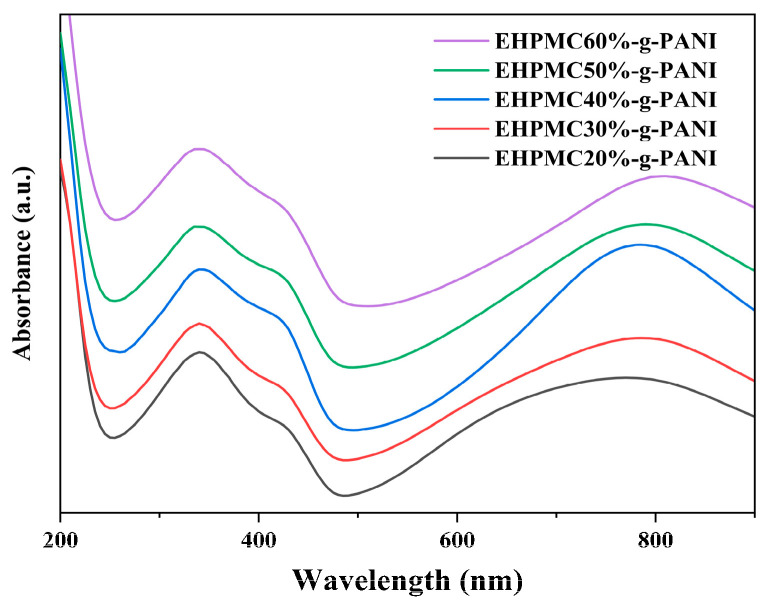
UV-vis spectra for EHPMC-g-PANI dispersions with different ECIP contents.

The UV-vis spectra for the EHPMC-g-PANI dispersions with different ECIP contents are presented in Figure 5. The figure reveals three characteristic absorption bands at 320–340 nm, 420–440 nm, and 750–820 nm, corresponding to the π-π* transition in the benzene ring, the polaron-π* transition, and the π-polaron transition, respectively [32]. These features are consistent with the structural characteristics of PANI. As the amount of ECIP increases, the intensity of the three main absorption bands initially increases and then decreases. The intensity of PANI’s absorption peaks are related to its degree of conjugation, with higher conjugation leading to higher conductivity. Consequently, when the amount of ECIP is 40% of the HPMC weight, the absorption peak intensity and conductivity of the EHPMC-g-PANI reaches its maximum values. Furthermore, as the amount of ECIP increases, the characteristic absorption band at 750–820 nm exhibits a red shift. This phenomenon is attributed to the increase in epoxy groups in the HPMC molecular chains, which provide more reaction sites for the aniline. Consequently, the particle size of the EHPMC-g-PANI dispersion gradually increases, leading to the observed red shift.

**Figure 6:**
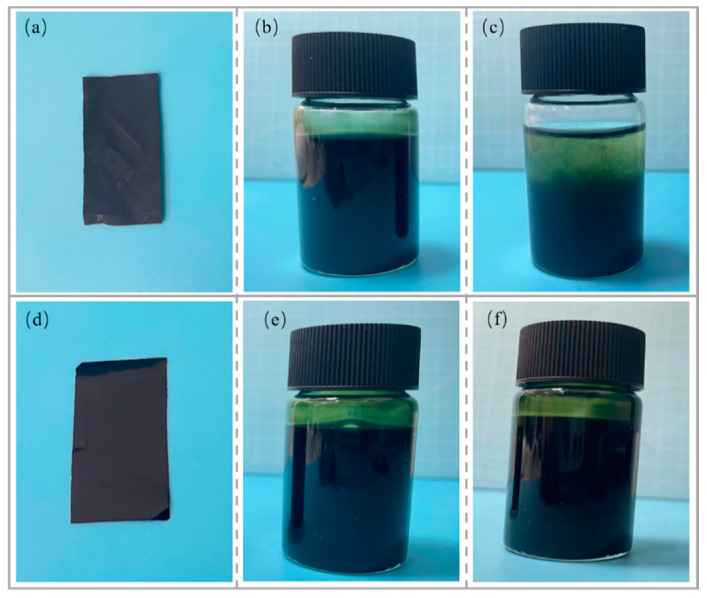
(**a**) HPMC/PANI composite conductive film; (**b**) HPMC/PANI dispersion (new); (**c**) HPMC/PANI dispersion (1 month); (**d**) EHPMC-g-PANI composite conductive film; (**e**) EHPMC-g-PANI dispersion (new); and (**f**) EHPMC-g-PANI dispersion (3 months).

The composite conductive films and dispersion diagrams for the HPMC/PANI and EHPMC-g-PANI are presented in Figure 6. The HPMC/PANI film exhibited a brown color with a visible granular texture and uneven distribution on the surface. The HPMC/PANI dispersion was yellow-green in color, and the dispersion stored for one month showed significant stratification, which was caused by the settling of the PANI particles. In contrast, the EHPMC-g-PANI film displayed a dark-green color with a smooth and even surface, devoid of any granular texture. Moreover, the EHPMC-g-PANI dispersion exhibited excellent uniformity and remained stable for three months at room temperature without any sedimentation. This demonstrates the superior storage stability of the EHPMC-g-PANI dispersion compared to the HPMC/PANI dispersion.

**Figure 7:**
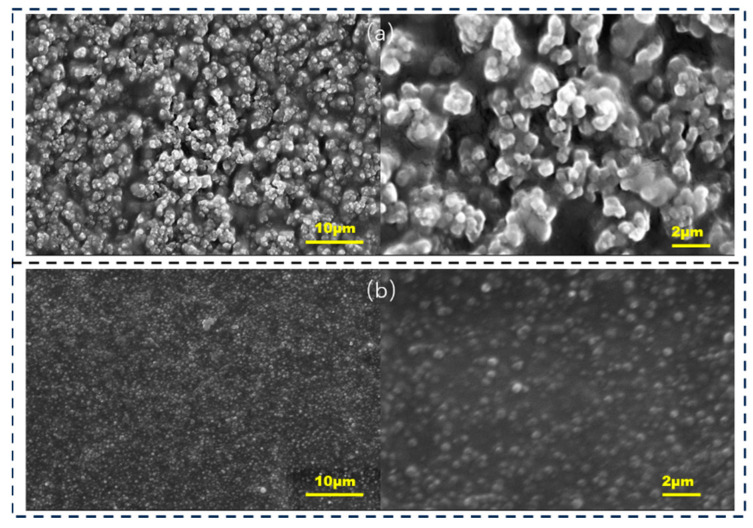
SEM images for (**a**) in situ polymerized HPMC/PANI composite conductive thin films and (**b**) EHPMC-g-PANI (ECIP-2 g) composite conductive thin films.

The SEM images for the HPMC/PANI and EHPMC-g-PANI films are presented in Figure 7. The HPMC/PANI film exhibited a rough surface morphology with a significant presence of irregularly dispersed spherical particles, and exhibited a size range of approximately 700–800 nm. These particles were attributed to the self-polymerization of PANI within the HPMC matrix. In contrast, the EHPMC-g-PANI film displayed a more compact and uniform particulate layer on its surface, with a notable reduction in particle size to approximately 200–300 nm. This improved morphology was attributed to the introduction of ECIP, which enhanced the compatibility between the HPMC and PANI, and facilitated a more homogenous integration of the two phases.

**Figure 8:**
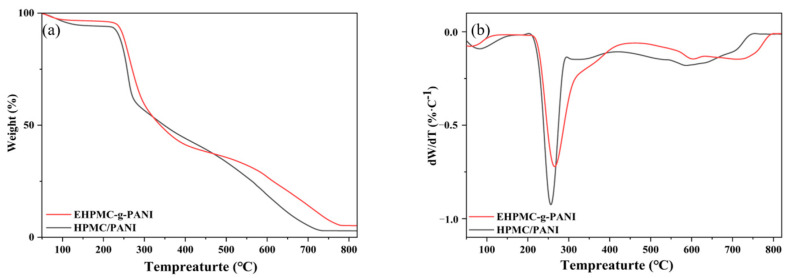
(**a**) TG and (**b**) DTG profiles for HPMC/PANI and EHPMC-g-PANI.

The TGA and DTG profiles for the HPMC/PANI and EHPMC-g-PANI are presented in Figure 8. The initial weight loss observed in the HPMC/PANI below 150 °C was attributed to the volatilization of the residual water and free HCl within the film. The second weight loss stage commenced at 200 °C, reaching its maximum rate at 256 °C. This was ascribed to the deprotonation of doped acid molecules from the PANI chains and the cleavage of the HPMC chains. The broader weight loss peak spanning 425–750 °C corresponded to the degradation and chain scission of the PANI. In comparison to the HPMC/PANI, the EHPMC-g-PANI exhibited a reduced rate of weight loss during the second stage, with the maximum rate occurring at 270 °C. Moreover, the onset of PANI chain scission and degradation was delayed to 470 °C and concluded at 798 °C. These findings indicate that the graft-polymerized EHPMC-g-PANI demonstrates significantly enhanced thermal stability compared to the in situ polymerized PANI/HPMC composite [32].

**Figure 9:**
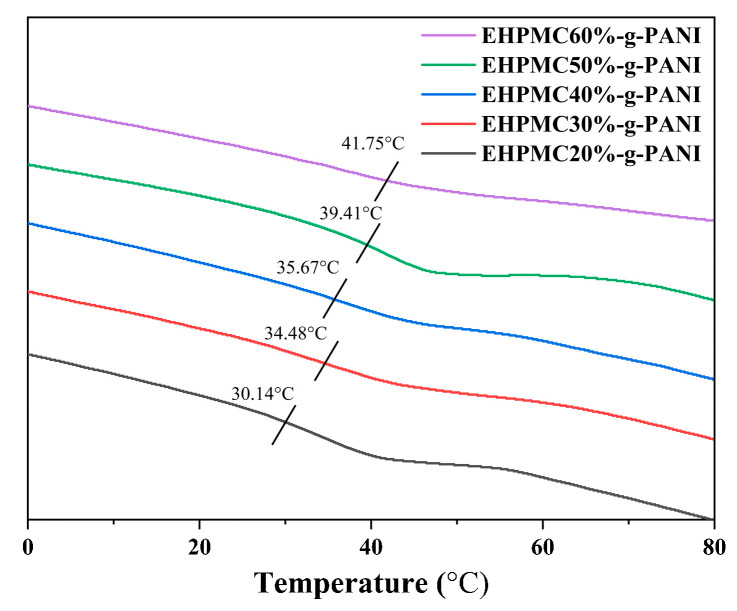
DSC for EHPMC-g-PANI composite conductive films with different ECIP contents.

The DSC for the EHPMC-g-PANI composite conductive films with varying ECIP contents are presented in Figure 9. A corresponding increase in the glass transition temperature (T_g_) of the EHPMC-g-PANI was observed from 30.14 °C to 41.75 °C when the amount of ECIP was increased from 20% to 60% of the HPMC weight. This phenomenon was attributed to the increasing grafting ratio of the epoxy groups with increasing ECIP content, which led to the incorporation of a greater amount of PANI into the HPMC molecular chains. The introduction of PANI enhanced the rigidity of the HPMC backbone and promoted intermolecular interactions, resulting in a reduction in the free volume and the restricted mobility of the molecular segments. Consequently, the EHPMC-g-PANI films required a higher energy input to reach the glass transition state, manifesting as an elevated T_g_.

**Figure 10:**
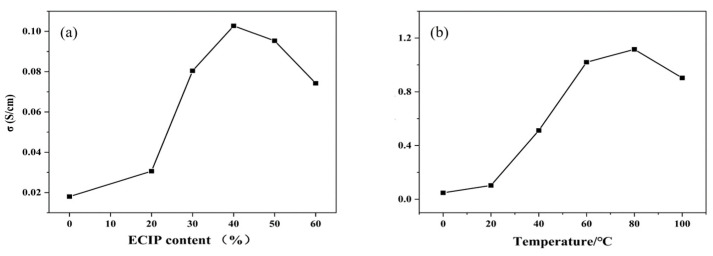
(**a**) Conductivity of EHPMC-g-PANI composite conductive films with different ECIP contents and (**b**) conductivity performance of EHPMC-g-PANI composite conductive films at different temperatures.

The influence of varying ECIP content on the conductivity of the EHPMC-g-PANI composite conductive films, as well as on the conductivity performance at different temperatures, are presented in Figure 10. Compared to the HPMC/PANI film prepared via in situ polymerization, which had a conductivity of only 0.018 S/cm, the conductivity of the EHPMC-g-PANI films increased significantly to 0.103 S/cm when the amount of ECIP was increased from 20% to 40% of the HPMC weight. However, a further increase in the ECIP content beyond 40% resulted in a decline in conductivity. Initially, the introduction of ECIP provided additional reactive sites for aniline grafting, effectively suppressing aniline self-polymerization and enhancing the compatibility between the PANI and HPMC phases. Consequently, more conductive pathways were formed within the EHPMC-g-PANI molecular chains, leading to an increase in conductivity. However, an excessive amount of ECIP resulted in shorter PANI chains, which are detrimental to the formation of efficient conductive pathways, ultimately causing a reduction in the film’s conductivity. A previous study by V. Cavalheiro Maeda et al. employed a blending method to fabricate a PANI-HPMC composite conductive film, which exhibited electrical conductivity in the range of 10^−3^–10^−2^ S/cm. In comparison, the EHPMC-g-PANI composite film synthesized in this study demonstrated a significant enhancement in conductivity, reaching an order-of-magnitude-higher value [33].

In addition, we also measured the conductivity changes in the films at different temperatures, which is important for their application in flexible electronic devices. The conductivity of the films exhibited an increasing trend with increasing temperature, reaching a peak value of 1.12 S/cm at 80 °C. However, when the temperature was further increased to 100 °C, the conductivity of the films decreased. The electrical conductivity of polyaniline is achieved through the movement of polarons along the polymer chain. As the temperature increases, the thermal energy of polyaniline molecules increases, promoting polaron hopping between adjacent molecular units. This increased hopping frequency contributes to the observed enhancement of electrical conductivity at elevated temperatures. Nevertheless, at sufficiently high temperatures, the electrical conductivity of polyaniline exhibits a decline. This phenomenon is attributed to the migration of the protonic acid dopant, which leads to a slight de-doping process.

**Figure 11:**
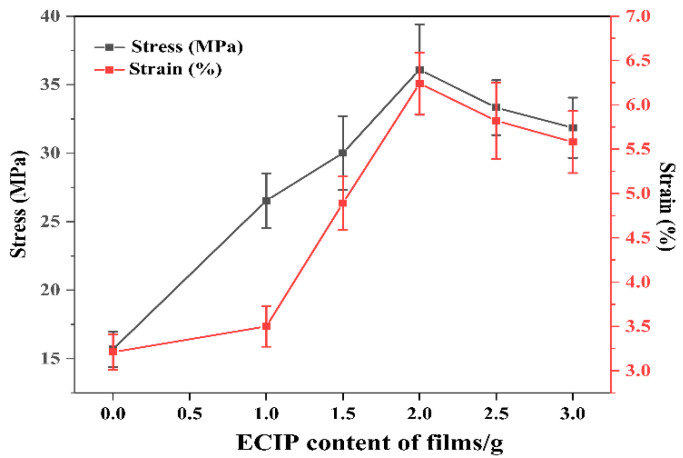
Tensile strength and elongation at break of EHPMC-g-PANI composite conductive films with different ECIP contents.

The effect of varying ECIP content on the tensile strength and elongation at break of the EHPMC-g-PANI films are presented in Figure 11. Doped PANI self-supporting films exhibit inherent brittleness and possess low strength and stiffness. In contrast, HPMC films demonstrate superior mechanical properties, exhibiting a tensile strength of 72.7 MPa and an elongation at break of 13.5%. Incorporating HPMC into the PANI system effectively enhances the mechanical properties of the composite conductive films. Compared to the HPMC/PANI film prepared via in situ polymerization, which exhibits a tensile strength and elongation at break of 15.69 MPa and 3.21%, respectively, the EHPMC-g-PANI films show significant improvement in both tensile strength and elongation at break, reaching 36.1 MPa and 6.24%, respectively, with 40% ECIP loading. However, a further increase in ECIP content beyond 40% leads to a decline in both the tensile strength and elongation at break. A suitable amount of ECIP effectively enhances the compatibility between the two phases, leading to the improved mechanical properties of the composite material. However, excessive ECIP content promotes the formation of an excessive crosslinking network within the conductive film, restricting the mobility of the polymer chains and ultimately reducing both the tensile strength and elongation at break.

## 5. Conclusions

This work presented an effective approach for fabricating conductive self-supporting films based on PANI and HPMC. By grafting PANI onto HPMC molecular chains, the challenge of PANI’s difficult processability was overcome, resulting in composite materials that combine the electrical conductivity of PANI with the excellent mechanical properties of HPMC. The introduction of epichlorohydrin significantly enhanced the compatibility between the two materials, leading to the improved overall properties of the composite material. The TGA analysis revealed a substantial increase in thermal stability. Notably, the composite exhibited a remarkable conductivity enhancement, from 0.018 S/cm to 0.103 S/cm, compared to that of the in situ polymerization of HPMC/PANI. Additionally, the tensile strength increased from 15.69 MPa to 36.1 MPa, and the elongation at break improved from 3.21% to 6.24%. The PANI and HPMC composite conductive films prepared in this work hold promising potential for applications in various fields, including flexible sensors, electronic components, and conductive inks.

## Data Availability

The original contributions presented in the study are included in the article, further inquiries can be directed to the corresponding author.
